# Dysregulation of Parvalbumin Expression in the *Cntnap2−/−* Mouse Model of Autism Spectrum Disorder

**DOI:** 10.3389/fnmol.2018.00262

**Published:** 2018-08-02

**Authors:** Emanuel Lauber, Federica Filice, Beat Schwaller

**Affiliations:** Anatomy Unit, Section of Medicine, University of Fribourg, Fribourg, Switzerland

**Keywords:** parvalbumin interneurons, calcium-binding proteins, contactin-associated protein-like 2, autism spectrum disorder, hyperpolarization-activated cyclic nucleotide-gated channels, perineuronal nets

## Abstract

Due to the complex and heterogeneous etiology of autism spectrum disorder (ASD), identification of convergent pathways and/or common molecular endpoints in the pathophysiological processes of ASD development are highly needed in order to facilitate treatment approaches targeted at the core symptoms. We recently reported on decreased expression of the Ca^2+^-binding protein parvalbumin (PV) in three well-characterized ASD mouse models, *Shank1−/−*, *Shank3B−/−* and *in utero* VPA-exposed mice. Moreover, PV-deficient mice (*PV+/−* and *PV−/−*) were found to show behavioral impairments and neuroanatomical changes closely resembling those frequently found in human ASD individuals. Here, we combined a stereology-based approach with molecular biology methods to assess changes in the subpopulation of PV-expressing (Pvalb) interneurons in the recently characterized contactin-associated protein-like 2 (*Cntnap2−/−*) knockout mouse model of ASD. The *CNTNAP2* gene codes for a synaptic cell adhesion molecule involved in neurodevelopmental processes; mutations affecting the human *CNTNAP2* locus are associated with human ASD core symptoms, in particular speech and language problems. We demonstrate that in *Cntnap2−/−* mice, no loss of Pvalb neurons is evident in ASD-associated brain regions including the striatum, somatosensory cortex (SSC) and medial prefrontal cortex (mPFC), shown by the unaltered number of Pvalb neurons ensheathed by VVA-positive perineuronal nets. However, the number of PV-immunoreactive (PV^+^) neurons and also PV protein levels were decreased in the striatum of *Cntnap2−/−* mice indicating that PV expression levels in some striatal Pvalb neurons dropped below the detection limit, yet without a loss of Pvalb neurons. No changes in PV^+^ neuron numbers were detected in the cortical regions investigated and also cortical PV expression levels were unaltered. Considering that Cntnap2 shows high expression levels in the striatum during human and mouse embryonic development and that the cortico-striato-thalamic circuitry is important for speech and language development, alterations in striatal PV expression and associated (homeostatic) adaptations are likely to play an important role in *Cntnap2−/−* mice and, assumingly, in human ASD patients with known Cntnap2 mutations.

## Introduction

The etiology of autism spectrum disorder (ASD) is highly complex and diverse. A combination of genetic, epigenetic and environmental factors is assumed to ultimately lead to the ASD phenotype characterized by impairments in social interaction and language/communication, and the presence of restricted or stereotyped patterns of behavior (Kleijer et al., 2014). The genetic architecture of ASD is extremely heterogeneous and difficult to apprehend. Common, rare, and *de novo* mutations are known to contribute to the genetic risk of ASD and the number of risk genes is steadily increasing (Bourgeron, 2015). Amongst the identified ASD risk genes, contactin-associated protein-like 2 (Cntnap2), coding for a synaptic cell adhesion molecule, is listed as a “strong candidate (2S)" in the Simons Foundation Autism Research Initiative database (SFARI)^1^. The *CNTNAP2* gene is one of the largest genes in humans spanning 2.3 Mb of genomic DNA, thus with an increased probability of structural rearrangements such as copy number variations (CNVs), insertions or deletions, single nucleotide polymorphisms (SNPs), altered transcription factor binding and epigenetic modifications affecting the *CNTNAP2* locus (Poot, 2015). Several studies identified CNVs, common and rare SNPs and/or polymorphisms in the *CNTNAP2* locus associated with syndromic neurodevelopmental disorders such as cortical dysplasia-focal epilepsy (CDFE) syndrome (Strauss et al., 2006), Pitt-Hopkins syndrome (Zweier et al., 2009), Gilles de la Tourette syndrome (Belloso et al., 2007), intellectual disability (ID) (Mikhail et al., 2011), obsessive compulsive disorder (Verkerk et al., 2003), attention deficit hyperactivity disorder (ADHD) (Elia et al., 2010), schizophrenia (Friedman et al., 2008) and autism (Alarcon et al., 2008; Arking et al., 2008). Although patients with *CNTNAP2* mutations display a complex phenotype, most of these patients manifest autistic characteristics; language impairments such as dysarthric language, language delay or absent speech/language are particularly frequent (Rodenas-Cuadrado et al., 2014; Penagarikano et al., 2015). Accordingly, *Cntnap2−/−* mice show impairments in social behavior and reduced vocalizations (Penagarikano et al., 2011; Brunner et al., 2015; Liska et al., 2017), deficits in learning and memory (Rendall et al., 2016) and epileptiform activity (Penagarikano et al., 2011; Thomas et al., 2016).

Due to the complex and heterogeneous etiology of ASD, major efforts are aimed to possibly identifying convergent pathways across different ASD mouse models in order to facilitate treatment approaches. One cell type, whose function is consistently impaired in different ASD mouse models (Wöhr et al., 2015) and post-mortem brains of human ASD patients (Hashemi et al., 2016) and thus representing a promising point-of-convergence with respect to ASD heterogeneity is a subset of GABAergic interneurons expressing parvalbumin (PV); hereafter termed Pvalb neurons. PV is a slow-onset Ca^2+^ buffer (more precisely described as a neuronal Ca^2+^ signal modulator) and a characteristic marker for fast-spiking interneurons (FSI). The number of Pvalb neurons (solely based on PV immunohistochemistry) was reported to be decreased in the cortex, striatum and hippocampus of *Cntnap2−/−* mice compared to WT mice (Penagarikano et al., 2011). However, we have shown in several validated ASD mouse models that the previously described “loss” of Pvalb neurons is caused by the down-regulation of PV in some neurons below a detectable threshold, while the number of Pvalb neurons is unchanged (Filice et al., 2016; Lauber et al., 2016). This is of great functional importance and was consistently found in *PV+/−*, *PV−/−*, *Shank1−/−*, *Shank3B−/−* and *in utero* VPA-exposed mice (VPA) mice; the genetic ASD mouse models also listed in the SFARI database. Here we show that the number of Pvalb neurons, identified by Vicia Villosa Agglutinin-positive (VVA^+^) perineuronal nets (PNN), is unchanged in *Cntnap2−/−* compared to WT mice in the somatosensory cortex, medial prefrontal cortex and striatum, three brain regions often implicated in ASD pathophysiology. However, decreased protein expression of PV was detected exclusively (selectively) in the striatum of *Cntnap2−/−* mice, as before in *Shank3B−/−* and VPA mice (Filice et al., 2016; Lauber et al., 2016), further supporting the hypothesis that PV down-regulation represents a common molecular endpoint across different ASD models. These findings thus closely link the *Cntnap2−/−* model with other previously validated ASD mouse models such as *Shank3B−/−* and VPA mice.

## Materials and Methods

### Animals

Mice were group-housed at the University of Fribourg, Switzerland, in temperature-controlled animal facilities (24°C, 12:12 h light/dark cycle). Mice had free access to water and were fed *ad libitum*. C57Bl/6J (WT), B6.129(Cg)-Cntnap2^tm1Pele^/J (*Cntnap2−/−*), B6.129-Shank3^tm2Gfng^/J (*Shank3B−/−*) mice were purchased from Jackson Laboratory (Bar Harbor, ME, United States). B6.Pvalb^tm1Swal^ (*PV−/−*) mice were generated by homologous recombination as described before (Schwaller et al., 1999). All strains were backcrossed to C57Bl/6J for more than ten generations and therefore considered congenic with C57Bl/6J. Thus, alterations at the level of morphology, biochemistry and molecular biology are assumed to be the result of genotypic differences and not related to mouse strain. Pups obtained from at least 3 different litters per group were used in order to exclude a litter bias. Mice were euthanized either with Esconarkon (300 mg/kg body weight; Streuli Pharma AG, Uznach, Switzerland) for subsequent perfusion, or by cerebral dislocation, if followed by dissection and isolation of selected brain regions. Only male mice participated in the experiments. All experiments were executed with the permission of the local animal care committee (Canton of Fribourg, Switzerland) and according to the present Swiss law and the European Communities Council Directive of 24 November 1986 (86/609/EEC).

### Tissue Preparation and Immunohistochemistry

Mice were anesthetized and perfused using 0.9% NaCl solution followed by 4% paraformaldehyde (PFA). Brains were removed and post-fixed in 4% PFA at RT for 24 h before they were transferred to 30% sucrose-TBS 0.1 M, pH 7.3 solution for cryopreservation at 4°C. After cryopreservation, brains were cut coronally in rostro-caudal direction using a freezing microtome (Frigomobil, Reichert-Jung, Vienna, Austria). Following stereological systematic random sampling principles, every sixth section was collected. Floating sections were then blocked for 1 h at RT in TBS 0.1 M, pH 7.3 containing 10% bovine serum albumin (BSA) and 0.4% Triton X-100. Afterward, sections were rinsed 3 times in TBS before being incubated using a PV antibody (anti-rabbit PV25, Swant, Marly, Switzerland) diluted 1:1000 and Vicia Villosa Agglutinin (biotinylated-VVA, Reactolab, Servion, Switzerland) at 10 μg/ml in TBS containing MgCl_2_, MnCl_2_, CaCl_2_ (final salt concentration: 0.1 mM each) for 16 h at 4°C. Next, sections were washed once in TBS and twice in Tris-HCl 0.1 M, pH 8.2 before incubation with the following antibodies: anti-rabbit Cy3-conjugated (diluted 1:200) and Cy2 streptavidin-conjugated (diluted 1:200, Milan Analytic AG, Switzerland) in Tris-HCl for 2 h at RT protected from light. After rinsing, sections were stained with DAPI (diluted 1:1000; LuBio Science GmbH, Luzern, Switzerland) in PBS 0.1 M, pH 7.4. After final rinsing, sections were mounted and cover-slipped using Hydromount mounting medium (National Diagnostics, Atlanta, GA, United States).

### Stereological Quantification and Counting Criteria

A Stereo Investigator system (Version 11, MicroBrightField, Williston, VT, United States) coupled to a Zeiss Axioplan microscope carrying a motorized x-y stage (Ludl Electronic Products, Ltd, Hawthorne, NY, United States) and connected to a Hamamtsu Orca Camera was used. Estimation of the total number of PV-positive (PV^+^) and Vicia Villosa Agglutinin-binding (VVA^+^) cells in pre-defined brain regions of interest (ROIs) was performed using the optical fractionator method (West et al., 1991) as described previously (Lauber et al., 2016). Stereotactic coordinates provided by the Paxinos and Franklin atlas (Paxinos, 2001) were used to define ROIs. The striatum (caudate-putamen) was defined at 1.10 to −0.82 mm from bregma, the SSC at 1.94 to −1.82 mm from bregma and the mPFC at 1.94 to 1.10 mm from bregma. Cell counting was performed on images obtained with the following objective lenses: 100x; NA = 1.40, oil immersion for the SSC; and 63x; NA = 1.30, oil immersion for the mPFC and striatum. The Cavalieri estimator (Gundersen et al., 1988) was used to estimate the total volume of the analyzed ROI. 6 WT mice and 5 *Cntnap2−/−* mice including pups obtained from at least three different litters per group were analyzed. All stereological parameters and results are reported in **Tables 1**, **2**, respectively.

**Table 1:** Stereological sampling parameters.

Brain region	Cutting plane	No. of sections	Section evaluation interval (μm)	Height of disector (μm)	Guard zone (μm)	Counting frame area (μm)	Sampling grid area (μm)	Measured section thickness mean (μm)	Cavalieri grid size (μm)
Striatum	coronal	6–7	6	20.0	0.5	110 × 90	350 × 350	21.61	150 × 150
SSC	coronal	14–15	6	20.0	0.5	70 × 55	450 × 450	21.35	200 × 200
mPFC	coronal	4	6	20.0	0.5	90 × 70	150 × 200	20.55	100 × 100

**Table 2:** Mean total number of PV^+^ and VVA^+^ cells in the striatum, SSC and mPFC of WT and *Cntnap2−/−* mice.

	Mean	*SD*	*P*-value	CE_m=0_/_m=1_ ≤	Mean	*SD*	*P*-value	CE_m=0_/_m=1_ ≤
	**Striatum PV^+^**				**Striatum VVA^+^**			
WT	21’298	948		0.10/0.06	26’413	486		0.10/0.06
KO	17’260	1’033	0.0001	0.13/0.08	26’461	2’241	0.9637	0.11/0.06
		
	**SSC PV^+^**				**SSC VVA^+^**			
WT	122’203	4’497		0.07/0.06	146’108	9’824		0.06/0.05
KO	118’656	8’590	0.4386	0.07/0.05	152’001	7’832	0.3259	0.06/0.05
		
	**mPFC PV^+^**				**mPFC VVA^+^**			
WT	6’572	248		0.11/0.07	7’170	645		0.10/0.07
KO	6’920	801	0.3359	0.12/0.07	7’350	801	0.6892	0.12/0.07

**FIGURE 1:**
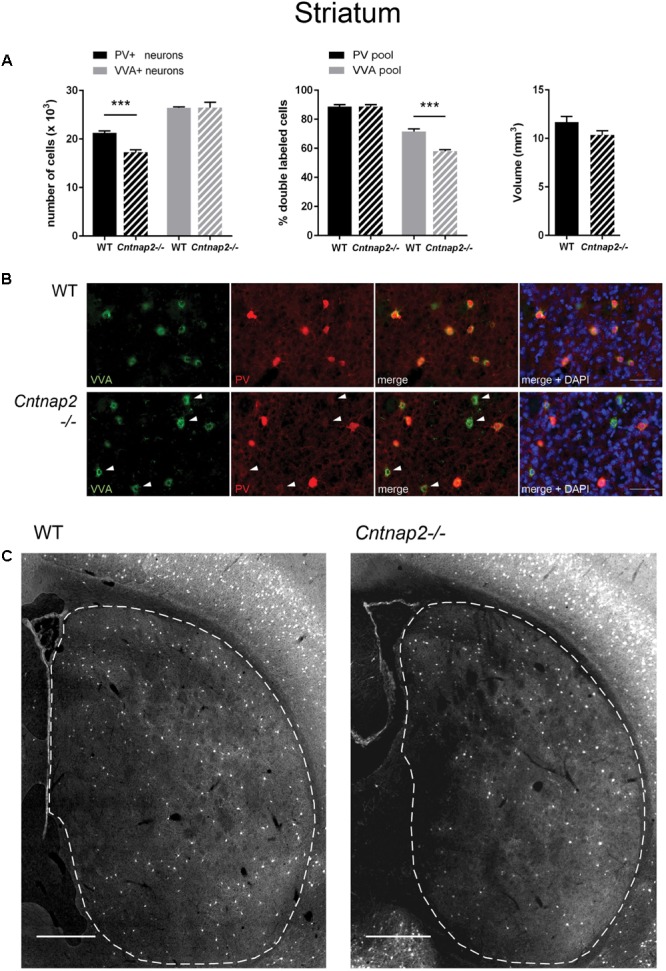
**(A)** Left: Stereological estimations of PV^+^ (black) and VVA^+^ (gray) cells in the striatum of PND25 WT (*N* = 6) and *Cntnap2−/−* (*N* = 5) mice. Middle: percentage of PV+ cells surrounded by VVA (black) and VVA+ cells showing PV expression (gray) in WT and KO mice. Right: Volume of the ROI; i.e., the striatum. Asterisks represent ^∗∗∗^*P* < 0.001. **(B)** Representative immunofluorescence images of PV (red) and VVA (green) co-localization from PND25 mouse striatum are shown. Less VVA^+^ cells are also positive for PV in *Cntnap2−/−* compared to WT mice (outlined by arrowheads). **(C)** Representative PV immunofluorescence images from the striatum of a WT and *Cntnap2−/−* mouse. ROIs are indicated with dashed lines (sections are located at Bregma 0.38 and 0.14 mm, respectively). Scale bar: **(B)** 50 μm; **(C)** 500 μm. All data are expressed as mean ± SEM.

PV^+^ and VVA^+^ cells were quantified independently and according to the following criteria: (1) presence of a DAPI-stained nucleus; (2) well-defined and roundish perineuronal net (PNN) for VVA^+^ cells; (3) nucleus-surrounding PV staining for PV^+^ neurons. Examples are shown in **Figure 1B**. At every fifth sampling location, the section thickness was measured and the mean of all measurements was used for computations. The total number of cells (N) in a defined ROI was estimated as proposed by West et al. (1991, 1996) using the formula:

$$N=\sum Q^{-}\times (1/ssf)\times (1/asf)\times (1/tsf)$$

where Q^−^ stands for “tops” (counts), and asf, tsf, and ssf for area sampling fraction, thickness sampling fraction and section sampling fraction, respectively. The coefficient of error CE was used to evaluate the precision of the estimates (Gundersen et al., 1999). CEs (*m* = 1 and *m* = 0) are provided in **Table 2**. To ensure independent and unbiased cell estimates, PV^+^ or VVA^+^ cells were quantified without cross-checking the other channel.

### RT-qPCR

After euthanasia by cervical dislocation, the whole brain of mice was quickly removed and put into ice-cold 0.9% NaCl solution for dissection. After cutting the brain in half along the midline, the cerebellum was removed. Next, the hippocampus followed by the striatum were dissected by carefully removing (pulling) the tissue pieces as described previously (see Figure S2 in Lauber et al., 2016). Unlike in our previous studies, exclusively cortical tissue was obtained by removing the remaining parts of the brain such as thalamus and fiber tracts from the cortex. Tissue samples were separately snap-frozen using liquid nitrogen before storing them at −80°C. Since RNA is more prone to degradation, the left hemisphere of the brain was dissected first and afterward used for RT-qPCR analysis. RNA was extracted from tissue pieces with the peqGold TRIzol reagent (Peqlab, VWR International GmbH, Erlangen, Germany). Promega’s Reverse Transcription Kit (Promega, Dübendorf, Switzerland) was used to synthesize cDNA. RT-qPCR to examine mRNA expression levels for *Gapdh, Rn18S, Pvalb, Gad67, Hcn1, Hcn2, Hcn4, Kcnc1, Kcnc2, and Kcns3* genes was performed using the universal 2X KAPA SYBR FAST qPCR Master Mix (Axonlab AG, Mont-sur-Lausanne, Switzerland) and a DNA thermal cycler (Corbett Rotor gene 6000, QIAGEN Instruments AG, Hombrechtikon, Switzerland). Primer sequences and PCR products are reported in **Table 3**. After initial denaturation at 95°C for 3 min, a two-step protocol was run: 40 cycles comprising denaturation at 95°C for 3 s and annealing/extension/data acquisition between 54 and 60°C for 20 s. The housekeeping genes *Gapdh* (striatum) and *Rn18S (*18S rRNA) (cortex) were used to normalize the mRNA content for each sample. Finally, the 2^−^^ΔΔCt^ method was used to quantify mRNA levels and genes of interest were normalized to I) the housekeeping gene and II) the control, i.e., wild-type group as previously described (Livak and Schmittgen, 2001).

**Table 3:** RT-qPCR primers.

Primer	Sequence 5′ – 3′	Nt position	Gene	Gene accession number
18S rRNA	For: TCAAGAACGAAAGTCGGAGGTT	1026–1047	*Rn18S*	NR_003278
	Rev: GGACATCTAAGGGCATCACAG	1493–1513		
GAD67	For: AATCTTGCTTCAGTAGCCTTCG	2979–3000	*Gad1*	NM_001312900
	Rev: TGTCTTCAAAAACACTTGTGGG	3178–3199		
GAPDH	For: ACCACAGTCCATGCCATCAC	612–631	*Gapdh*	NM_001289726
	Rev: CACCACCCTGTTGCTGTAGCC	1041–1061		
HCN1	For: CTCAGTCTCTTGCGGTTATTACG	1138–1160	*Hcn1*	NM_010408
	Rev: TGGCGAGGTCATAGGTCAT	1210–1228		
HCN2	For: ATCGCATAGGCAAGAAGAACTC	1936–1957	*Hcn2*	NM_008226
	Rev: CAATCTCCTGGATGATGGCATT	2017–2037		
HCN4	For: GCATGATGCTTCTGCTGTGT	1268–1287	*Hcn4*	NM_001081192
	Rev: GCTTCCCCCAGGAGTTATTC	1371–1390		
K_v_3.1	For: GTGCCGACGAGTTCTTCTTC	1362–1381	*Kcnc1*	NM_001112739
	Rev: GTCATCTCCAGCTCGTCCTC	1646–1665		
K_v_3.2	For: AGATCGAGAGCAACGAGAGG	72–91	*Kcnc2*	NM_001025581
	Rev: GGTGGCGATCGAAGAAGAAT	379–398		
K_v_9.3	For: CCCTGGACAAGATGAGGAAC	465–484	*Kcns3*	NM_173417
	Rev: TTGATGCCCCAGTACTCGAT	745–764		
Pvalb	For: TGTCGATGACAGACGTGCTC	24–43	*Pvalb*	NM_013645
	Rev: TTCTTCAACCCCAATCTTGC	309–328		

### Western Blot Analysis

Tissue samples from the right hemispheres were sonicated and soluble proteins extracted for Western blotting. Proteins (30 μg/sample) were separated under denaturing conditions by SDS-PAGE (12.5%). Following gel electrophoresis, semi-dry transfer was performed to transfer proteins onto nitrocellulose membranes (MS solution, Chemie Brunschwig, Basel, Switzerland). The membranes were initially blocked at RT for 1 h in 5% BSA in TBS 0.1 M, pH 7.6 followed by incubation with primary antibodies: rabbit anti-PV25 (Swant, Marly, Switzerland), rabbit anti-GAPDH (Sigma-Aldrich, Buchs, Switzerland) both diluted 1:10,000 in TBS with 0.1% Tween (TBS-T) and 2% BSA overnight at 4°C. On the next day, membranes were rinsed 3 times with TBS-T before being incubated for 1 h at RT in the secondary antibody containing solution: goat anti-rabbit IgG HRP-conjugated (Sigma–Aldrich, Buchs, Switzerland) diluted 1:10,000 in TBS-T. Finally, membranes were washed 3 times with TBS before development using ECL (Merck Millipore, Schaffhausen, Switzerland). Image Studio Light Version 5.0 software was used to quantify visualized bands. GAPDH bands served as loading control on all membranes.

### Statistical Analysis and Cell Number Estimates

A two-tailed, unpaired *t*-test was used to compare stereological data, mRNA and protein levels between the two groups. Data were analyzed using the R version 3.3.3 software. Stereological data from the two hemispheres of the same mouse were pooled and analyzed together. A *p*-value < 0.05 was considered to be statistically significant.

## Results

### Unaltered Numbers of Pvalb Neurons, but Decreased PV Expression Levels in the Striatum of *Cntnap2−/−* Mice

Unbiased stereological analysis of brain sections obtained from postnatal day (PND) 25 ± 1 control C57Bl/6J (WT) and *Cntnap2−/−* mice was aimed to obtain robust and precise cell number and volume estimates in different brain regions. The optical fractionator method and the Cavalieri estimator were used to quantify cell numbers and volumes in the striatum, somatosensory cortex (SSC) and medial prefrontal cortex (mPFC), three brain regions frequently implicated in ASD pathology. Coefficient of error (CE) values ranged from 0.05 to 0.11 for the WT group and from 0.05 to 0.13 for the *Cntnap2−/−* group; all stereological parameters and results are reported in **Tables 1**, **2**, respectively. We independently quantified the number of parvalbumin-positive (PV^+^) and Vicia Villosa Agglutinin-positive (VVA^+^) cells in the same regions of interest as described before (Filice et al., 2016; Lauber et al., 2016). VVA recognizes N-acetylgalactosamine residues on the surface of perineuronal nets (PNN), which preferentially surround Pvalb neurons (Hartig et al., 1992; Haunso et al., 2000). Thus, Pvalb neurons with strongly decreased PV expression levels (i.e., below the detectable threshold) or even completely devoid of PV, may still be detected by the VVA^+^ staining as previously shown in *PV+/−* and *PV−/−* mice, respectively (Filice et al., 2016). In line with previous findings in *Cntnap2−/−* mice that reported a ∼20–25% decrease in the number of PV^+^ neurons in the striatum, cortex and hippocampus at PND14 (Penagarikano et al., 2011), we found a significant decrease in the order of 20% (*p* = 0.0001) in the total number of PV^+^ cells in the striatum of PND25 *Cntnap2−/−* mice compared to WT mice. Yet the total number of VVA^+^ cells was unchanged between the two genotypes, indicating that there was no loss of Pvalb neurons in the striatum of *Cntnap2−/−* mice (**Figure 1A**). This conclusion was confirmed by the percentage of double-positive cells. As observed in WT mice, ∼90% of PV^+^ cells were also VVA^+^ in *Cntnap2−/−* mice; however, only ∼58% of cells out of the VVA pool were detected as PV^+^ cells in *Cntnap2−/−* mice (∼72% in WT), indicating that ∼15% (*p* = 0.0002) of VVA^+^ cells had “lost” their PV signal compared to WT mice (**Figure 1A**). Representative immunofluorescence images of VVA^+^, PV^+^ and double-positive cells in the striatum are shown in **Figures 1B,C**, respectively. The VVA^+^ immunofluorescence images corresponding to **Figure 1C** are shown in Supplementary Figure 1. It is evident that while almost all VVA^+^ cells co-localized with PV^+^ cells in the WT group resulting in red-fluorescent cells with a “yellow rim,” several VVA^+^ cells were PV-immuno-negative (“green only”) in the striatum of *Cntnap2−/−* mice (**Figure 1B**). The mean volume of the striatum, as determined using the Cavalieri estimator, was unchanged between the two genotypes (**Figure 1A**).

**FIGURE 2:**
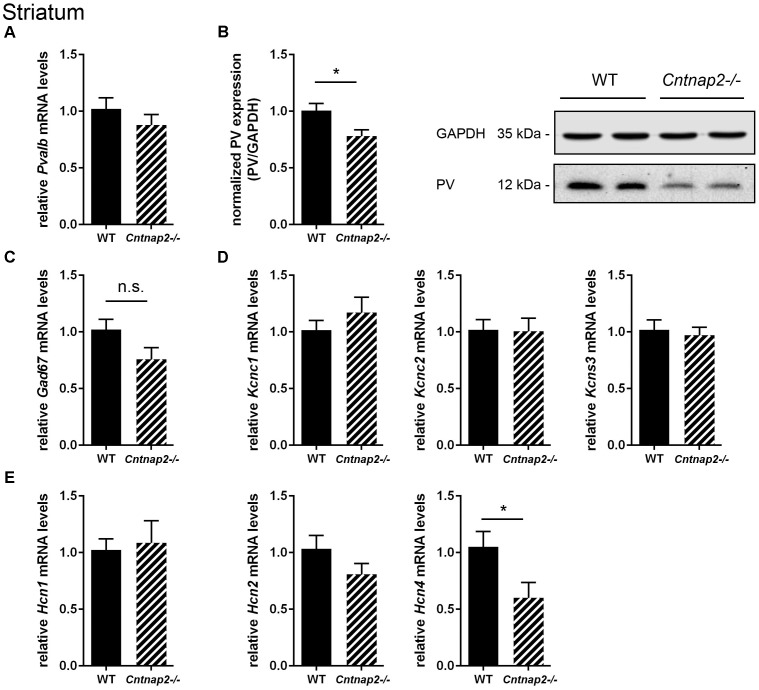
RT-qPCR values from striatal samples of PND25 mice representing mRNA levels for **(A)** *Pvalb*, **(C)** *Gad67*, **(D)** Left: *Kcnc1*; Middle: *Kcnc2*; Right: *Kcns3*, **(E)** Left: *Hcn1*; Middle: *Hcn2*; Right: *Hcn4*. Signals were normalized to *Gapdh* mRNA levels and expressed as fold change compared to WT (*N* = 6 mice each). **(B)** Left: Quantitative Western blot analysis of striatal samples of PND25 WT and *Cntnap2–/–* mice (*N* = 6 mice each). Quantification of PV protein levels in the striatum is shown. Asterisks represent ^∗^*P* ≤ 0.05. GAPDH signals served as loading controls and were used for the normalization of PV protein signals. Results are expressed as a percentage of normalized PV/GAPDH signals in the WT group. Right: Representative Western blot signals for PV and GAPDH are shown. All data are expressed as mean ± SEM.

RT-qPCR and Western blot analysis of striatal tissue revealed changes in PV protein expression levels between WT and *Cntnap2−/−* mice. The trend toward a decrease in *Pvalb* mRNA levels in *Cntnap2−/−* mice measured by RT-qPCR did not reach statistical significance compared to WT samples (∼10%, *p* = 0.3190) (**Figure 2A**). But more importantly at the functional level and consistent with the stereological analysis, Western blot analysis of *Cntnap2−/−* striatal lysates revealed a ∼22% (*p* = 0.0219) decrease in PV protein levels compared to WT (**Figure 2B**; uncropped images of all Western blots are shown in Supplementary Figure 2). Representative protein Western blot signals for PV and GAPDH are shown in **Figure 2B**. Taken together, these results confirm that *Cntnap2−/−* mice exhibit striatal PV down-regulation during early post-natal development without any indication for Pvalb neuronal loss. The non-significant decrease in *Pvalb* transcript levels hints toward a possible post-transcriptional regulation, unlike in the previously investigated ASD mouse models *Shank3B−/−* and VPA mice, where both *Pvalb* transcript and PV protein levels were decreased to a similar extent (Filice et al., 2016; Lauber et al., 2016).

To further support that the total number of striatal Pvalb neurons was unchanged between the two groups, mRNA levels for typical universal GABAergic interneuron markers and Pvalb neuron-specific markers were investigated. Glutamate decarboxylase isoform 67 *(Gad67)* mRNA levels were not significantly different between WT and *Cntnap2−/−* mice (**Figure 2C**). Of note, Gad67 is not exclusively expressed in Pvalb neurons, but also in other interneurons. Thus, additional Pvalb neuron-specific markers were analyzed including the potassium voltage-gated channels subfamily C member 1 (*Kcnc1*), the protein also known as K_v_3.1 (Chow et al., 1999) and subfamily S member 3 (*Kcns3*) coding for K_v_9.3 (Georgiev et al., 2012). Both potassium channel transcripts were found to be unaltered between WT and *Cntnap2−/−* mice (**Figure 2D**). The same held true for *Kcnc2* (K_v_3.2) (**Figure 2D**), the next closest relative of *Kcnc1*, which also co-localizes with Pvalb neurons but to a lesser extent than *Kcnc1* (Chow et al., 1999).

In an exploratory approach, we quantified mRNA levels for hyperpolarization-activated cyclic nucleotide-gated (HCN) channel isoforms 1, 2, and 4. HCN channel expression and/or the currents mediated by these channels (I_h_ current) were previously reported to be altered in cultured hippocampal neurons derived from *Shank3−/−* mice (Yi et al., 2016) and in VPA mice (Lauber et al., 2016), two validated mouse ASD models with a similar striatal PV deficit as the *Cntnap2−/−* mice. While *Hcn1* and *Hcn2* mRNA expression levels in *Cntnap2−/−* mice did not differ from transcript levels in WT mice in the striatum, *Hcn4* mRNA levels were significantly decreased by ∼40% (*p* = 0.0418) in *Cntnap2−/−* mice (**Figure 2E**). The conceivable functional implications of these results are discussed below.

### Lack of *Cntnap2* Has No Effect on the Number of Pvalb Neurons and PV Expression in SSC and mPFC

**FIGURE 3:**
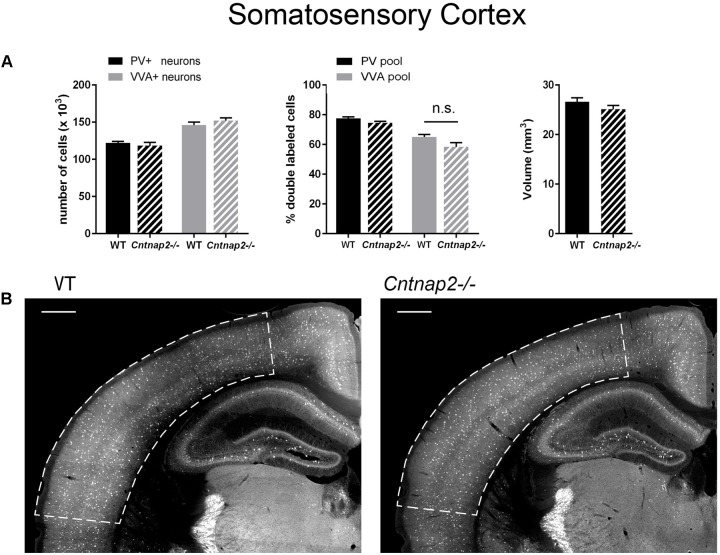
**(A)** Left: Stereological estimations of PV^+^ (black) and VVA^+^ (gray) cells in the SSC of PND25 WT (*N* = 6) and *Cntnap2–/–* (*N* = 5) mice. Middle: percentage of PV^+^ cells surrounded by VVA (black) and VVA^+^ cells showing PV expression (gray) in WT and *Cntnap2–/–* mice. Right: Volume of the ROI; i.e., the SSC. **(B)** Representative PV immunofluorescence images from the SSC of a WT and *Cntnap2–/–* mouse. ROIs are indicated with dashed lines (sections are located at Bregma –1.34 mm). Scale bar: 500 μm. All data are expressed as mean ± SEM.

**FIGURE 4:**
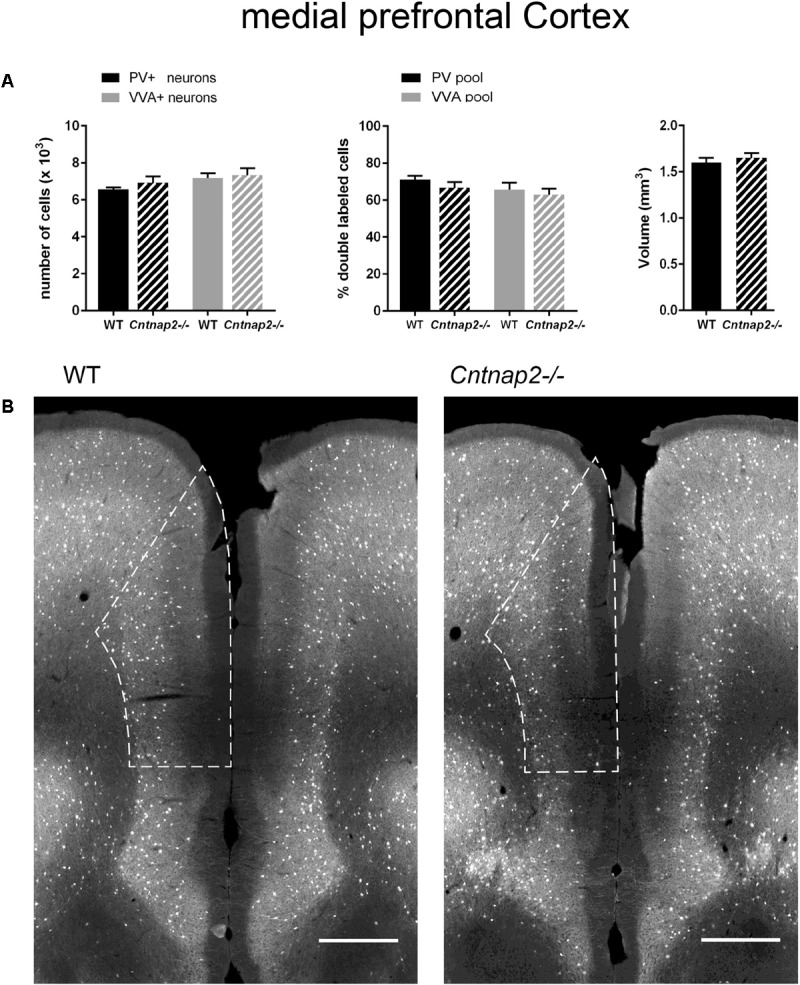
**(A)** Left: Stereological estimations of PV^+^ (black) and VVA^+^ (gray) cells in the mPFC of PND25 WT (*N* = 6) and *Cntnap2–/–* (*N* = 5) mice. Middle: percentage of PV^+^ cells surrounded by VVA (black) and VVA^+^ cells showing PV expression (gray) in WT and *Cntnap2–/–* mice. Right: Volume of the ROI; i.e., the mPFC. **(B)** Representative PV immunofluorescence images from the mPFC of a WT and *Cntnap2–/–* mouse. ROIs are indicated with dashed lines (sections are located at Bregma 1.70 mm). Scale bar: 500 μm. All data are expressed as mean ± SEM.

**FIGURE 5:**
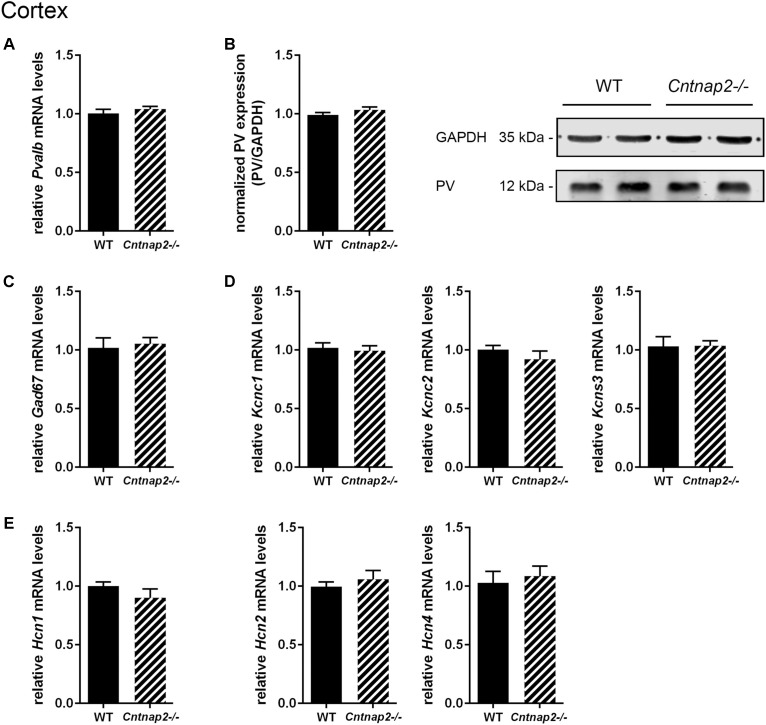
RT-qPCR values from cortical samples of PND25 mice representing mRNA levels for **(A)** *Pvalb*, **(C)** *Gad67*, **(D)** Left: *Kcnc1*; Middle: *Kcnc2*; Right: *Kcns3*, **(E)** Left: *Hcn1*; Middle: *Hcn2*; Right: *Hcn4*. Signals were normalized to *Rn18S* mRNA levels and expressed as fold change compared to WT (*N* = 6 mice each). **(B)** Left: Quantitative Western blot analysis of cortical samples of PND25 WT and *Cntnap2–/–* mice (*N* = 6 mice each). Quantification of PV protein levels in the cortex is shown. GAPDH signals served as loading controls and were used for the normalization of PV protein signals. Results are expressed as a percentage of normalized PV/GAPDH signals in the WT group. Right: Representative Western blot signals for PV and GAPDH are shown. All data are expressed as mean ± SEM.

Unlike previously reported by Penagarikano et al. (2011) and by Vogt et al. (2017) in the SSC of PND14 and PND30 *Cntnap2−/−* mice, respectively, we did not find Pvalb neuron-specific alterations in the cortex of the same mice at PND25. Neither were PV^+^ cell numbers altered in the SSC and mPFC, nor did we observe differences in the number of VVA^+^ cells in the same brain regions (**Figures 3A**, **4A**). Accordingly, the percentage of double-positive cells, in both directions (PV pool and VVA pool), was not significantly altered between WT and *Cntnap2−/−* mice in the SSC and mPFC (**Figures 3A**, **4A**). The volumes of the SSC and mPFC were also similar between groups (**Figures 3A**, **4A**). Representative immunofluorescence images of the SSC and mPFC are depicted in **Figures 3B**, **4B**, respectively. In agreement with the unchanged numbers of Pvalb neurons, subsequent mRNA and protein quantification showed that *Pvalb* transcript and PV protein levels were essentially identical between WT and *Cntnap2−/−* mice in cortical tissue obtained from male PND25 mice (**Figures 5A,B**). Similarly, PV protein levels in the hippocampus and cerebellum were not different between genotypes at PND25 (Supplementary Figure 3). RT-qPCR analysis of cortical tissue confirmed the stereological findings. No alterations in the mRNA levels for *Gad67*, *Kcnc1*, *Kcnc2*, and *Kcns3* were observed in cortical extracts from *Cntnap2−/−* mice at PND25 (**Figures 5C,D**). Concomitantly, mRNA levels for the three HCN family members were unchanged in the cortex of *Cntnap2−/−* mice (**Figure 5E**). We had previously reported about increased *Hcn1* mRNA levels in the cortex of VPA mice, which might thus explain some of the electrophysiological deficits that have previously been found in this ASD model (Rinaldi et al., 2007, 2008; Lauber et al., 2016).

### Unchanged Transcript Levels of Pvalb Neuron Markers *Kcnc1* and *Kcns3*, as Well as *Hcn* Family Members in Adult (PND70) *Cntnap2−/−* Mice

The role of Cntnap2 in the regulation and electrophysiological properties of Pvalb neurons had been investigated previously. Vogt et al. (2017) had used a cell transplantation approach, where neurons from the medial ganglionic eminence (MGE) of E13.5 *Cntnap2+/+*, *Cntnap2+/−* and *Cntnap2−/−* mice were transplanted into the cortex of PND1 WT mice. These tdTomato-tagged neurons gave rise also to Pvalb neurons evidenced by their fast, non-accommodating firing properties. After 6–8 weeks *in vivo*, several electrophysiological parameters were found to be different, depending on whether the MGE-derived Pvalb neurons were from *Cntnap2+/+* or *Cntnap2−/−* mice. In neurons from the latter, the resting membrane potential was more positive, the AP spike half-width increased, the maximum AP slope decreased, and also the input resistance was slightly increased; the authors had hypothesized that these changes might be caused by alterations in K^+^ and/or Na^+^ channels. Since changes related to K^+^ conductances had been observed in striatal neurons with reduced or absent PV expression (e.g., higher firing frequency, increased excitability, slower AHP recovery, more regular inter spike interval) (Bischop et al., 2012; Orduz et al., 2013), we performed Western blot analysis of cortical extracts obtained from adult mice (PND70 ± 1). However, we found no evidence for reduced PV levels in the cortex of PND70 *Cntnap2−/−* mice (Supplementary Figures 4A,B), as we had also observed in PND25 mice (**Figures 3–5**). Moreover, no changes in mRNA levels for *Pvalb, Kcnc1 Kcns3, Hcn1* and *Hcn4* were detected between WT and *Cntnap2−/−* mice in the cortex at PND70 (Supplementary Figures 4C,D). Of note, the previously reported differences were observed in the MGE-derived implanted neurons from WT and *Cntnap2−/−* mice and not between endogenous Pvalb neurons from the two genotypes and might thus explain the apparent discrepancies (see also Discussion).

### “Analogous” Changes in the Striatum of Mouse ASD Models (*Cntnap2−/−, PV−/−, Shank3B−/−*, VPA) May Hint Toward Points of Convergence

**FIGURE 6:**
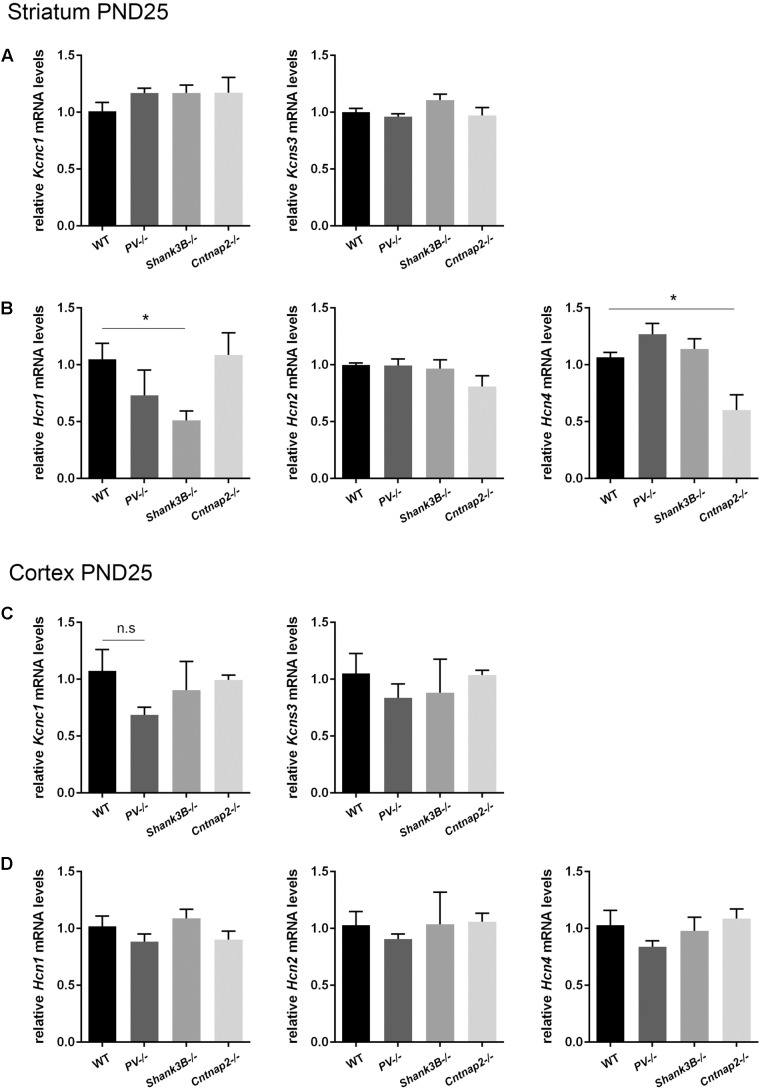
RT-qPCR values from striatal and cortical samples of WT, *PV–/–, Shank3B–/–*, and *Cntnap2–/–* PND25 mice representing mRNA levels for **(A)** and **(C)** Left: *Kcnc1*; Right: *Kcns3*, **(B)** and **(D)** Left: *Hcn1*; Middle: *Hcn2*; Right: *Hcn4*. Signals were normalized to *Gapdh* (striatum) or *Rn18S* (cortex) mRNA levels and expressed as fold change compared to WT (*N* = 5 mice each). All data are expressed as mean ± SEM.

Based on the hypothesis that various genetic and environmental ASD mouse models might entail similar alterations in the expression levels of proteins present in Pvalb neurons and/or in Pvalb neuron networks, transcript levels of the selective Pvalb neuron markers *Kcnc1* and *Kcns3*, and HCN family members (*Hcn1*, *Hcn2*, and *Hcn4*) in the striatum and cortex were quantified in *PV−/−* and *Shank3B−/−* mice and compared to results obtained in *Cntnap2−/−* mice, the latter also shown in **Figures 2D,E**). While *Kcns3* transcript levels were unchanged in both brain regions of *PV−/−*, *Shank3B−/−* and *Cntnap2−/−* mice compared to WT mice (**Figures 6A,C**) and thus supporting the previously drawn conclusion that there is no loss of Pvalb neurons in these ASD mouse models (Filice et al., 2016), there was a trend toward decreased expression of *Kcnc1* in the cortex of *PV−/−* mice (*p* = 0.0885; **Figure 6C**). A similar down-regulation of *Kcnc1* transcript and K_V_3.1 protein had been observed before in the cortex of VPA-exposed mice (Lauber et al., 2016). mRNA levels for *Hcn1*, *Hcn2* and *Hcn4* were unchanged in the cortex of all 3 ASD models (**Figure 6D**). Levels for *Hcn2* were also similar in the striatum of all investigated models; however *Hcn1* expression levels were prominently decreased in *Shank3B−/−* mice (∼50%, *p* = 0.0113). Although *Hcn1* signals were rather weak in the striatum, the finding of altered *Hcn1* levels in *Shank3B−/−* mice is of interest, since Hcn-mediated I_h_-channelopathy was previously suggested to be a main driver for the ASD phenotype observed in *Shank3−/−* mice (Yi et al., 2016) (see Discussion). In addition, *Hcn4* transcript levels were significantly lower in the striatum of *Cntnap2−/−* compared to WT mice (**Figure 6B**). While decreases in transcript levels of *Pvalb* and *Kcnc1* are in most cases also mirrored in lower protein expression levels (Filice et al., 2016; Lauber et al., 2016), it is currently unknown whether such a correlation also holds true for Hcn channels.

In summary, our results suggest that Pvalb-neuron numbers are not altered in the investigated cortical regions and moreover that global cortical PV expression levels in PND25 ± 1 *Cntnap2−/−* mice are unchanged. Yet in the striatum of Cntnap2-deficient mice, we found decreased PV protein expression levels. The implications of striatal PV down-regulation in the various ASD mouse models are discussed below.

## Discussion

The interest in PV-expressing (Pvalb) interneurons linked to neurodevelopmental disorders such as ASD and schizophrenia has steadily grown over the past years. The number of PV^+^ neurons was reported to be decreased in human ASD patients (Hashemi et al., 2016) and several ASD mouse models (see Table 1 in Wöhr et al., 2015). In most cases, this decrease was assumed to be the result of partial Pvalb neuron loss. Noteworthy, RNA-seq and RT-qPCR analyses on post-mortem tissue samples of temporal and frontal cortex as well as cerebellum from 48 ASD patients and 49 healthy controls showed that the *PVALB* gene was in the group of most strongly down-regulated genes in the ASD group (Parikshak et al., 2016). So far, mutations in the *PVALB* gene have not been reported in human ASD patients. Nonetheless, mice with decreased or absent PV levels (*PV+/−* and *PV−/−*) show a strong behavioral ASD phenotype and moreover, ASD-associated neuroanatomical changes (Wöhr et al., 2015). These findings support the hypothesis that PV may represent a promising molecular target providing a common endpoint for at least some forms of ASD.

Here, we showed that the number of PV^+^ cells is reduced in the striatum of *Cntnap2−/−* mice at PND25 ± 1. The time point was chosen, since PV expression and the electrophysiological maturation of Pvalb neurons reach adult levels at this age (de Lecea et al., 1995; Okaty et al., 2009). Additionally, at this age, mice with reduced or absent PV levels (PV+/−, PV−/−) manifest an ASD-like behavioral phenotype (Wöhr et al., 2015; Filice et al., 2018). The decrease in PV^+^ cells is the result of PV down-regulation and not due to a partial loss of Pvalb neurons, since the number of VVA^+^ cells serving as a proxy measure of striatal Pvalb neurons (Hartig et al., 1992) was unchanged between WT and *Cntnap2−/−* mice. Also transcript levels for specific and well-described Pvalb neuron markers such as K_V_3.1 (*Kcnc1*) and K_V_9.3 (*Kcns3*) were unaltered in the striatum of *Cntnap2−/−* mice. In order to understand the mechanisms and the functional consequences of Cntnap2-deficiency resulting in a behavioral ASD-like phenotype in mice, the distinction between Pvalb neuron loss *vs.* PV-downregulation is of utmost relevance. A “simple” loss of inhibitory interneurons such as the Pvalb subtype is expected to result in a decrease of the inhibitory tone in the brain. In contrast, Pvalb neurons with decreased or absent PV expression were consistently found to exhibit enhanced short-term facilitation (Schwaller, 2012; Orduz et al., 2013), hence increasing the inhibitory tone. Recently, by using fiber photometry, unperturbed Pvalb neuronal activity in the mPFC of freely moving *Cntnap2−/−* and WT mice was recorded, when mice were confronted with different social and non-social stimuli (Selimbeyoglu et al., 2017). Distinct activity patterns were observed in the two groups in response to the different stimuli, suggesting that the underlying neural dynamics of Pvalb neurons in the mPFC differed between genotypes. Additionally, expression of stabilized step-function opsin (SSFO) in Pvalb neurons allowed to optogenetically increasing Pvalb neuron excitability in the mPFC, which rescued the social deficits prevailing in *Cntnap2−/−* mice and was associated with reduced activity of cortico-striatal projections. This finding is in line with the hypothesis that reduced PV levels are another mean of enhancing the output of PV neurons in order to increase the inhibitory tone in the brain, according to the notion of homeostatic plasticity (Turrigiano, 2011). Interestingly, human patients carrying mutations affecting the *CNTNAP2* locus and adult (>6 month) *Cntnap2−/−* mice display frequent and severe seizures (Strauss et al., 2006; Penagarikano et al., 2011), a state generally linked to an altered excitation/inhibition balance. Optogenetically enhancing Pvalb neuron excitability (i.e., enhancing inhibition) shown to rescue social deficits in *Cntnap2−/−* mice (Selimbeyoglu et al., 2017), might also have an effect on seizure severity, yet this remains to be proven experimentally. Of note, in PND25 *Cntnap2−/−* mice, we found no indication of cortical PV down-regulation, thus cortical alterations in Pvalb neuron excitability –if present– may not be linked to PV expression; whereas the reduction of PV levels in striatal Pvalb neurons likely increases excitability as shown before in the same neuron population in *PV−/−* mice (Orduz et al., 2013). Striatal PV downregulation was found to be a common feature in several prototypical and well-established mouse ASD models. In addition to the present findings in *Cntnap2−/−* mice, also in *Shank3B−/−* and *in utero* VPA-exposed mice, reduced PV levels (in the order of ∼25–50%) and unaltered numbers of Pvalb neurons were reported (Filice et al., 2016; Lauber et al., 2016). This suggests that in these ASD models, the striatum is a particularly susceptible region and that it might play an important role in the development/manifestation of ASD core symptoms in mice. The basal ganglia, where the striatum serves as input structure, have developed to a complex circuitry in higher vertebrates not only mediating motor output control, but also higher cognitive functions such as sensory processing, learning, and memory tasks as well as the generation of intentional and motivated behaviors in a reward-guided manner (Fuccillo, 2016). There is substantial clinical evidence that striatal dysfunction is associated with ASD. Several MRI studies reported about alterations in the volume of the caudate nucleus or putamen (Langen et al., 2007, 2014; Estes et al., 2011) and enhanced striatal connectivity in ASD patients compared to healthy controls (Di Martino et al., 2011). Abnormalities in striatal structure and function have moreover been described for several ASD mouse or rat models, including *Shank3−/−* mice (Peca et al., 2011; Jaramillo et al., 2016) and *in utero* VPA-exposed rats (Schneider et al., 2007). Recently, it was demonstrated that targeted depletion of PV-expressing fast-spiking interneurons (FSIs) and large cholinergic interneurons (CINs) specifically in the dorsal striatum of mice leads to spontaneous stereotypy and marked impairments in social interactions (Rapanelli et al., 2017).

Currently, reports about *Cntnap2−/−* mice in relation to ASD are still sparse. Cntnap2 is a member of the neurexin family of synaptic cell adhesion molecules involved in the clustering of K+ channels in myelinated axons (Poliak et al., 1999, 2003). However, since myelination takes place postnatally and Cntnap2 is strongly expressed during embryonic development, Cntnap2 is expected to fulfill additional functions in early brain development. This assumption is reinforced by the rising number of reports linking mutations in the *CNTNAP2* locus with ASD (Rodenas-Cuadrado et al., 2014). Three independent studies reported about reduced spine density in neurons derived from Cntnap2-deficient mice relative to controls, associated with a decrease in synaptic transmission (Anderson et al., 2012; Gdalyahu et al., 2015; Varea et al., 2015). Also perisomatic evoked IPSCs were found to be significantly reduced in hippocampal slices of *Cntnap2−/−* mice (Jurgensen and Castillo, 2015).

During human and mouse embryonic development, Cntnap2 shows enriched expression in the frontal lobe, striatum and thalamus (Abrahams et al., 2007; Alarcon et al., 2008). Within the cortex, Cntnap2 is particularly abundant in certain interneuron subpopulations: in chandelier cells and PV^+^ neurons, as well as in VIP (CR^+^ and CCK^+^) cells (Vogt et al., 2017). The lack of Cntnap2 leads to a decrease in the number of PV^+^ neurons, which was interpreted as a defect in the differentiation and/or activity of Pvalb neurons resulting secondarily in decreased PV expression (Vogt et al., 2017) evidenced at PND14. In line, a delayed maturation of Pvalb neurons associated with impaired PV circuit function in ASD has been reported before, e.g., in *Shank3−/−* mice (Gogolla et al., 2014). As mentioned before, the cortico-striato-thalamic circuitry is crucially involved in higher order cognitive functions and Cntnap2 appears to be particularly important for language development, since most human patients with Cntnap2 mutations manifest some sort of language impairment (see Table 1 in Penagarikano et al., 2015). Compared to WT mice, *Cntnap2−/−* mice emit less ultrasonic vocalizations in the isolation-induced pup vocalization test (Penagarikano et al., 2011), as well as in response to the female urine stimulus (Brunner et al., 2015). Interestingly, Cntnap2 is also highly expressed within the cortico-striato-thalamic circuitry of the zebra finch songbird, a popular animal model for vocal learning (Panaitof et al., 2010). Lesioning of the lateral magnocellular nucleus of the anterior neostriatum in songbirds results in songs with monotonous repetitions of a single note complex (Scharff and Nottebohm, 1991), thus providing an additional connection between striatal Cntnap2 expression deficits and language-related impairments. Taken together, decreased/absent Cntnap2 expression and/or striatal impairments seem to be potent inducers of ASD core symptoms, with language dysfunction being particularly frequent in patients affected by mutations in the *CNTNAP2* gene. The observed down-regulation of PV in the striatum of *Cntnap2−/−* mice may thus represent an adaptive mechanism to counteract reduced inhibition, as observed in the hippocampus of these mice (Jurgensen and Castillo, 2015), and thus keeping the excitation/inhibition balance within a correct physiological window.

Striatal PV down-regulation during early postnatal development does not seem to be the only similarity between *Cntnap2−/−* mice, *Shank3B−/−* and VPA-exposed mice. Using human and mouse neurons, Yi et al. (2016) demonstrated that mutations in Shank3 predispose to ASD possibly by inducing a severe I_h_-channelopathy. Hyperpolarization-activated cation (I_h_) currents, which are mediated by hyperpolarization-activated cyclic nucleotide-gated (HCN) channels, regulate membrane resting potentials, input resistance, neuronal excitability and synaptic transmission (Biel et al., 2009). Neurons derived from *Shank3−/−* mice were found to exhibit severely decreased I_h_ currents and furthermore, decreased Hcn4 protein levels *in vitro* (Yi et al., 2016). When quantifying mRNA levels of the *Hcn* isoforms 1, 2 and 4 in the cortex and striatum of *Cntnap2−/−* mice, we found reduced mRNA levels of *Hcn4* exclusively in the striatum of *Cntnap2−/−* mice compared to WT animals, while mRNA levels of the other isoforms were unchanged in both brain regions. Therefore, putative impairments in Hcn-mediated I_h_ currents might also contribute to the phenotype of *Cntnap2−/−* mice. Of note, the observed changes in striatal *Hcn4* transcript levels are but a first hint toward possible alterations in the striatum of these mice. Whether Hcn4 protein levels are altered, in which neuron population and to which extend I_h_ currents are functionally altered in *Cntnap2−/−* mice, remains to be thoroughly investigated.

In summary, we show that *Cntnap2−/−* mice display down-regulation of PV expression in the striatum, a brain structure with enriched Cntnap2 expression during early development and functional importance for higher cognitive functions in later life. Striatal PV down-regulation moreover seems to occur in various ASD mouse models including *Cntnap2−/−*, *Shank3B−/−*, *in utero* VPA-exposed (and evidently *PV−/−*) mice. Moreover, striatal PV down-regulation was associated with a decrease in *Hcn* isoform transcripts in *Cntnap2−/−* and *Shank3B−/−* mice, representing another point of possible convergence in ASD-implicated mechanisms.

## Author Contributions

BS devised the study and took part in data analysis and manuscript writing. EL carried out all the experiments, did the statistical analysis and took part in writing the manuscript. FF participated in setting up the experiments and writing the manuscript. All of the authors read and endorsed the final version of the manuscript.

## Conflict of Interest Statement

The authors declare that the research was conducted in the absence of any commercial or financial relationships that could be construed as a potential conflict of interest. The reviewer MJS and handling Editor declared their shared affiliation at time of review.

## Footnotes

1 https://gene.sfari.org
