# Supplementary material for: Dysregulation of Parvalbumin Expression in the Cntnap2−/− Mouse Model of Autism Spectrum Disorder
Source: Front Mol Neurosci. 2018 Aug 2;11:262. doi: 10.3389/fnmol.2018.00262 (PMC6082962; doi:10.3389/fnmol.2018.00262)
Supplement: Supplementary file 1 [file Presentation_1.pdf]

Supplementary Material

Dysregulation of parvalbumin expression in the Cntnap2-/- mouse model of autism spectrum disorder

Emanuel Lauber[1], Federica Filice[1], Beat Schwaller[1*]

[1]Anatomy Unit, Section of Medicine, University of Fribourg, Route Albert-Gockel 1, CH-1700 Fribourg, Switzerland

*** Correspondence:**
Prof. Dr. Beat Schwaller,
Anatomy, Department of Medicine, University of Fribourg,
Route Albert-Gockel 1, CH-1700 Fribourg, Switzerland.
Phone: ++41 26 300 85 08 ; Fax: ++41 26 300 97 33
Email: Beat.Schwaller@unifr.ch

Supplementary Figures

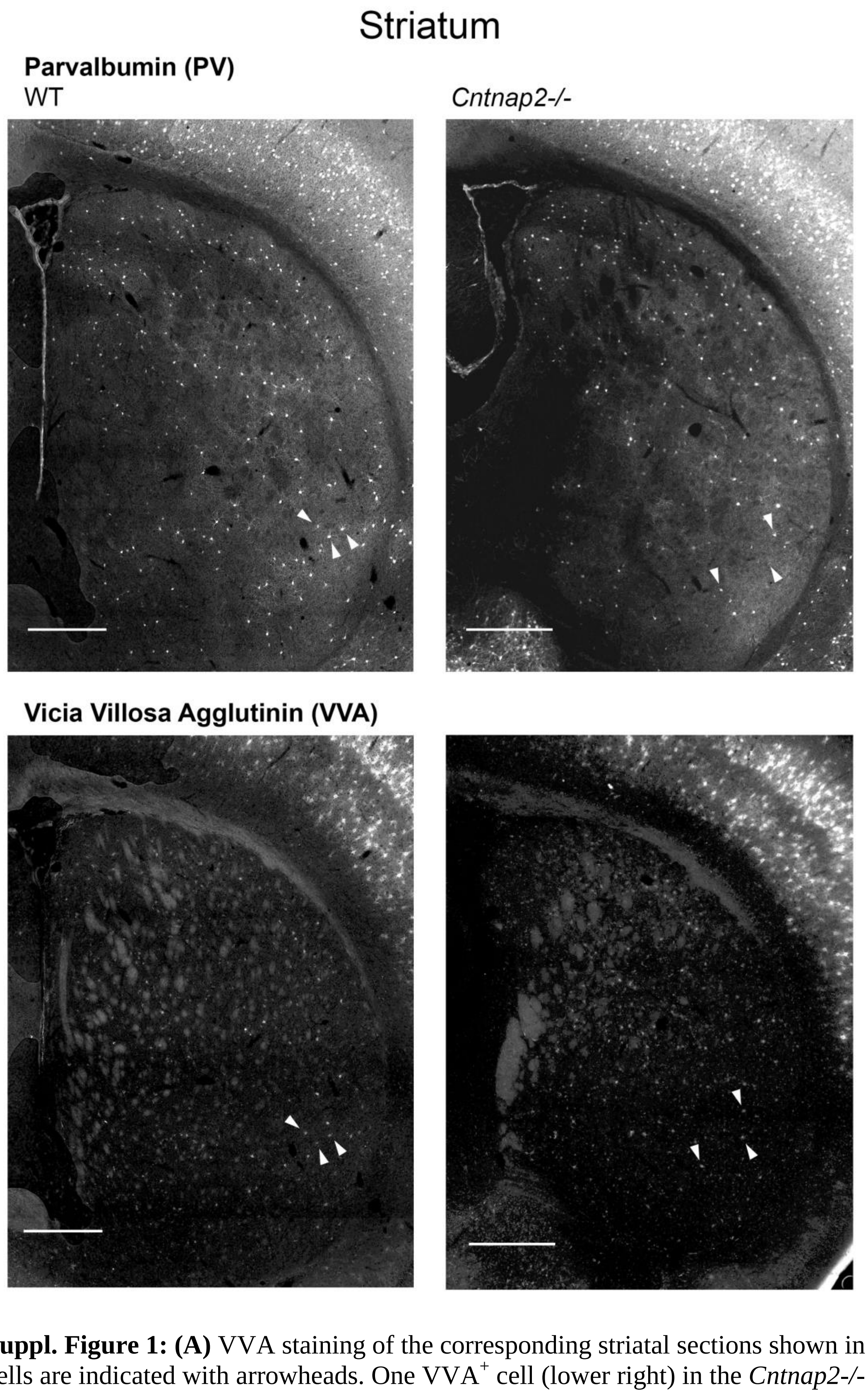

Suppl. Figure 1: (A) VVA staining of the corresponding striatal sections shown in Figure 1. VVA^+ cells are indicated with arrowheads. One VVA^+ cell (lower right) in the *Cntnap2-/-* group appears to be negative for PV. Scale bar 500 µm.

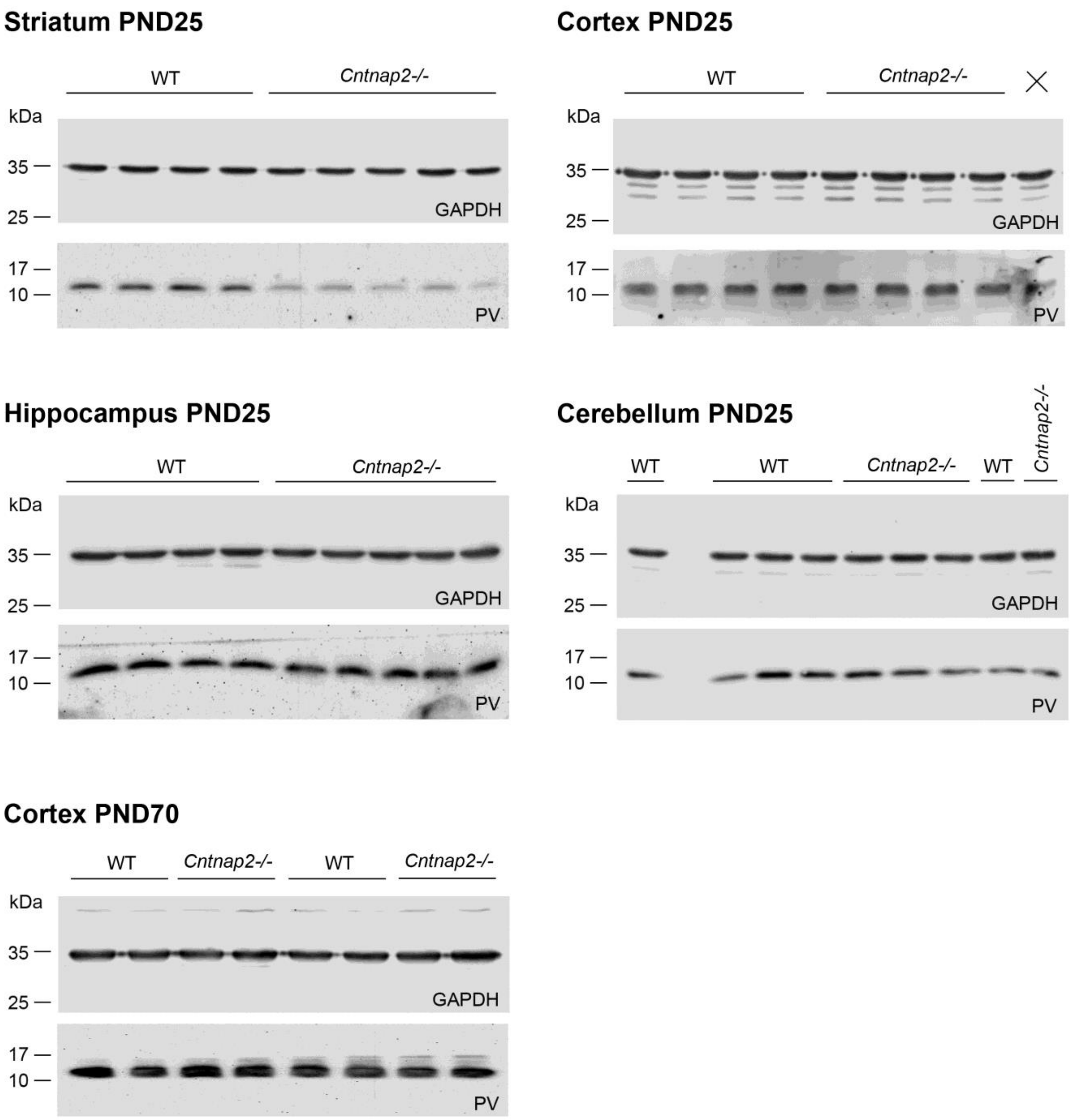

Suppl. Figure 2: Complete uncropped images of the Western blots shown in the manuscript.

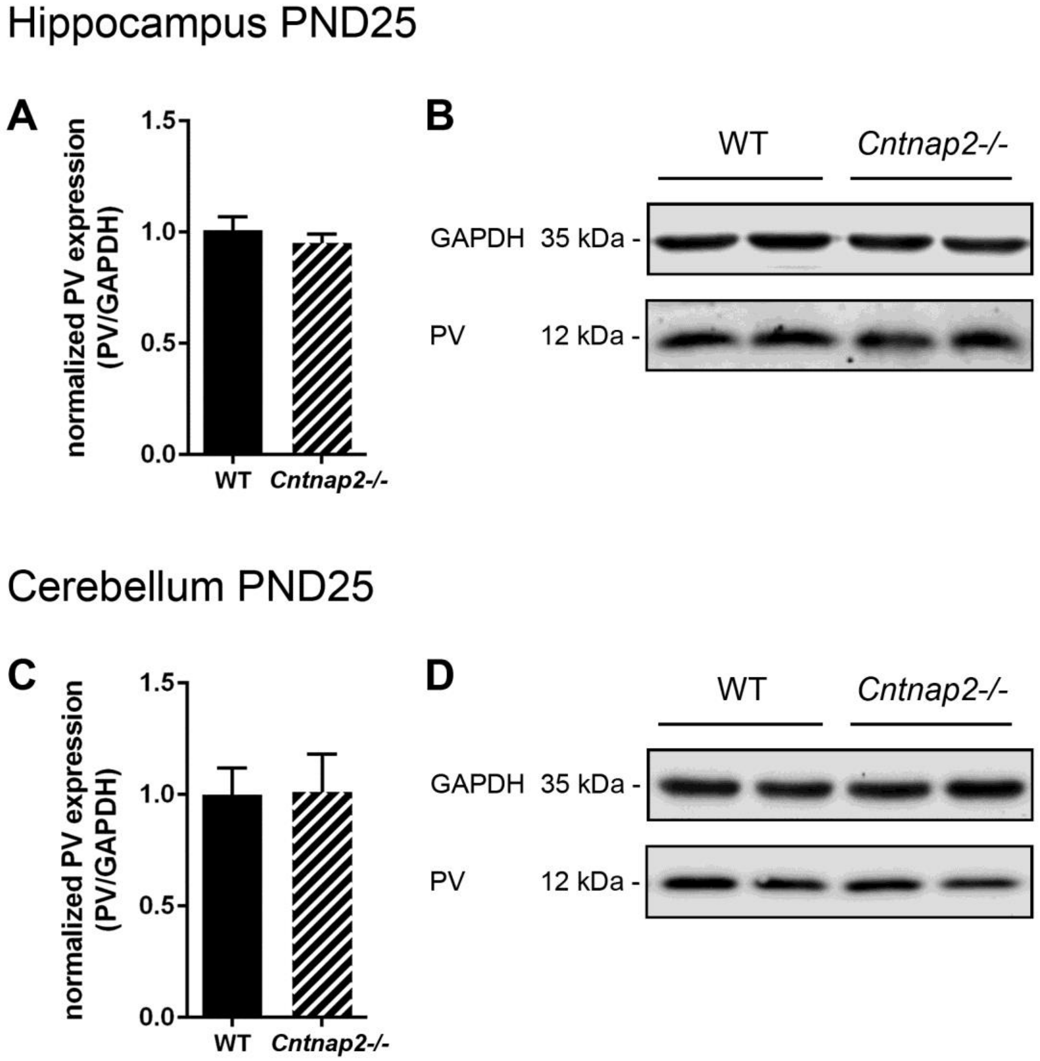

Supplementary Figure 3: Quantitative Western blot analysis of **(A)** hippocampal and **(C)** cerebellar samples of PND25 WT and *Cntnap2-/-* mice (N = 5 mice each). Quantification of PV protein levels is shown. GAPDH signals served as loading controls and were used for the normalization of PV protein signals. Results are expressed as a percentage of normalized PV/GAPDH signals in the WT group. Representative Western blot signals for PV and GAPDH are shown for the **(B)** hippocampus and **(D)** cerebellum. All data are expressed as mean ± SEM.

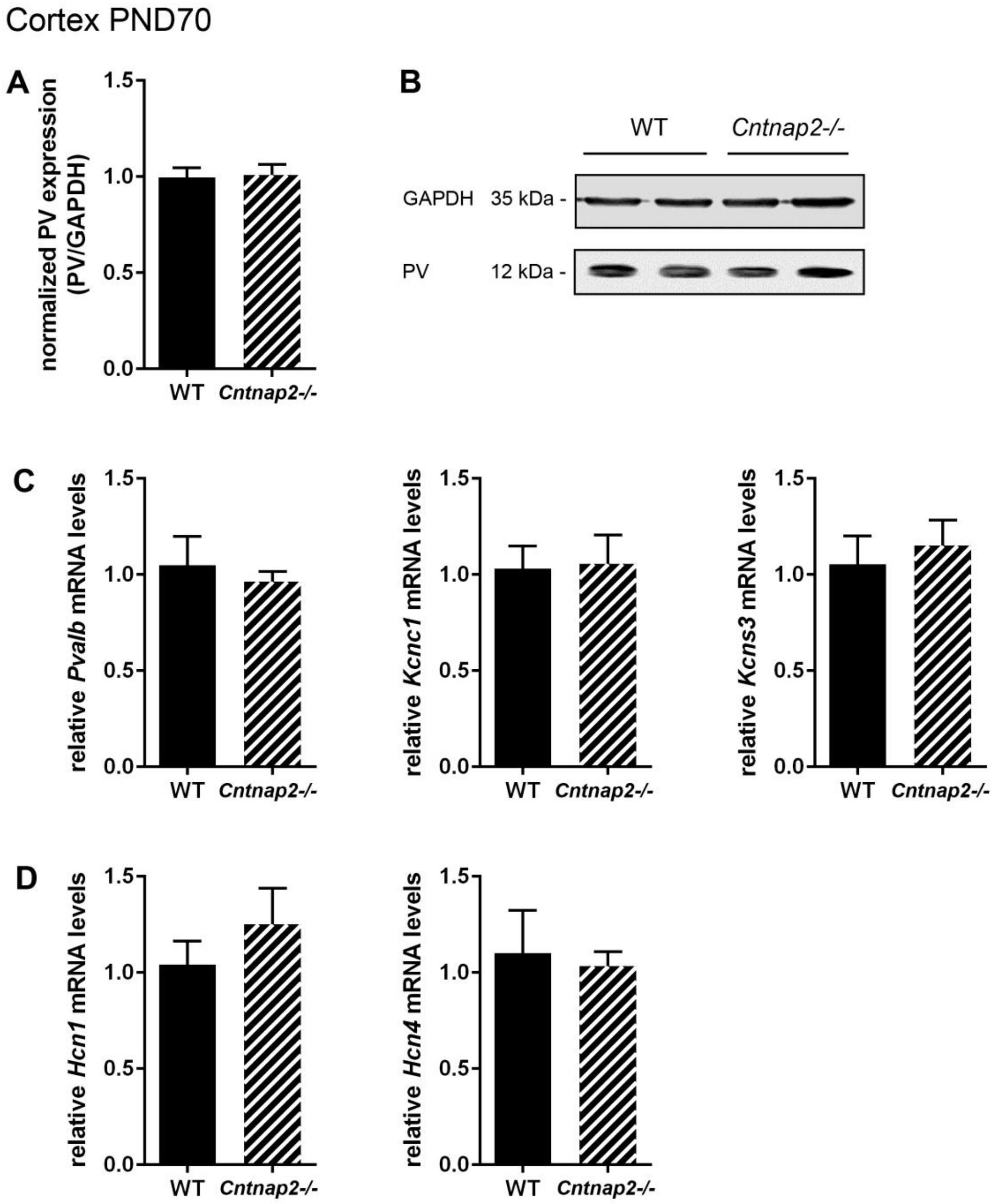

Suppl. Figure 4: (A) Quantitative Western blot analysis of cortical samples of PND70 WT and *Cntnap2-/-* mice (N = 5 mice each). Quantification of PV protein levels is shown. GAPDH signals served as loading controls and were used for the normalization of PV protein signals. Results are expressed as a percentage of normalized PV/GAPDH signals in the WT group. **(B)** Representative Western blot signals for PV and GAPDH are shown. **(C and D)** RT-qPCR values from cortical samples of WT and *Cntnap2-/-* PND70 mice representing mRNA levels for **(C) Left**: *Pvalb*; **Middle:** *Kcnc1*; **Right**: *Kcns3*, **(D) Left**: *Hcn1*; **Right**: *Hcn4*. Signals were normalized to *Rn18S* mRNA levels and expressed as fold change compared to WT (N = 5 mice each). All data are expressed as mean ± SEM.
